# Strategic health policy and health system resilience in protracted crisis settings: policymaker perspectives from Yemen

**DOI:** 10.1080/16549716.2026.2734009

**Published:** 2026-09-25

**Authors:** Dalia Hyzam, Taha Al-Mahbashi, Fekri Dureab, Timo Ulrichs

**Affiliations:** a Institute for Research in International Assistance (IRIA), Akkon University, Berlin, Germany; b Women Research and Training Center (WRTC), University of Aden, Aden, Yemen; c Faculty of Medicine and Health Sciences, Al-Razi University, Sana’a, Yemen; d College for Health Sciences, National University College for Health Sciences, Sana’a, Yemen; e Department of Healthcare Management, College of Business, University of Doha for Science and Technology, Doha, Qatar

**Keywords:** Institutional learning, service continuity, donor dependence, emergency financing, referral governance

## Abstract

**Background:**

Health system resilience is often described through the World Health Organization building blocks, yet in protracted crises it depends on whether governance, financing, workforce, information systems, service delivery, and accountability can convert crisis response into institutional reform.

**Objective(s):**

This study examines how far Yemen’s health system responses to prolonged conflict, economic decline, disease outbreaks, and COVID-19 progressed beyond absorptive and adaptive resilience toward transformative resilience, and how strategic health policy across the building blocks shaped this transition.

**Methods:**

We conducted 30 in-depth interviews with national and subnational policymakers and health leaders in Yemen between May and July 2025. Data were analysed in ATLAS.ti 25 using framework-informed thematic analysis that combined a priori categories from the World Health Organization health system building blocks and resilience capacities with inductively developed empirical codes.

**Results:**

Yemen demonstrated externally buffered absorptive capacity sufficient to prevent complete system collapse, together with uneven and localised adaptation through crisis committees, donor-supported financing, temporary workforce mobilisation, intensified surveillance, oxygen and isolation capacity, and referral workarounds. However, these responses were largely reactive, donor-dependent, and unevenly institutionalised. Transformative resilience remained constrained by fragmented authority, under-protected recurrent financing, workforce instability, weak data-to-decision pathways, limited referral governance, and the absence of formal learning-to-reform mechanisms.

**Conclusions:**

Yemen’s experience shows that resilience in protracted crisis settings should be judged not only by the survival of repeated shocks, but by whether absorptive and adaptive gains are converted into institutional capability through strategic policy alignment across the health system.

## Paper Context

**Main findings**: Yemen demonstrated externally buffered absorptive resilience and limited adaptive responses that helped prevent complete system collapse, but transformative resilience remained constrained by fragmented governance, unprotected recurrent financing, workforce instability, weak data-to-decision pathways, and absent institutional learning-to-reform mechanisms.

**Added knowledge**: This study provides rare policy-relevant empirical evidence from Yemen showing that crisis coping and partial adaptation can coexist with blocked transformation, while distinguishing between maintaining service continuity under shock and converting crisis response into durable institutional reform.

**Global health impact for policy and action**: Strengthening resilience in conflict-affected health systems requires moving beyond emergency surge responses toward durable policy anchors, including clarified decision rights, protected operations and maintenance financing, regulated surge workforce mechanisms, integrated information systems linked to decision-making, and formal learning-to-reform cycles.

## Background

Health system resilience is frequently discussed through the World Health Organization (WHO) health system building blocks: leadership and governance, financing, workforce, medical products and technologies, information systems, and service delivery [1]. However, resilience does not arise from strengthening components in isolation; it depends on the strategic health policy architecture that aligns authority, financing, regulation, coordination, information use, and accountability. Resilience literature commonly distinguishes absorptive capacity, adaptive capacity, and transformative capacity [2–4]. In this analysis, strategic health policy architecture refers to the configuration of policy instruments and decision rules covering decision rights, financing protection, regulatory enforcement, coordination mechanisms, information use, and accountability that links the building blocks rather than treating them as separate components. This emphasis reflects a wider shift in the resilience literature away from a purely structural reading of the building blocks and toward a relational, complex adaptive systems perspective that foregrounds feedback loops, self-organisation, learning, and institutional adaptation [5–8].

Yemen is a demanding context for examining resilience. Since 2015, conflict, economic contraction, recurrent disease outbreaks, and COVID-19 have unfolded simultaneously. In 2025, 19.5 million people required humanitarian assistance and protection [9]. Since 2017, operational visibility has expanded through the WHO’s deployment of the Health Resources and Services Availability Monitoring System (HeRAMS), with national-scale reporting on facility functionality and service availability in 2023 and 2024 [10,11]. Yet peer-reviewed studies have documented persistent geographic inequities in access to functional facilities and significant declines in routine hospital service utilisation during the early waves of COVID-19. These findings suggest that improved reporting and operational visibility alone are insufficient to secure continuity of care; rather, resilience depends on how health policy translates information into coordinated governance, financing, and service delivery decisions during periods of sustained stress [12,13].

Before 2014, the Yemeni health system was already fragile, characterised by chronic underinvestment, heavy out-of-pocket spending, workforce shortages, and uneven coverage between urban and rural areas [14,15]. Governance was centralised but institutionally weak, with limited regulatory enforcement and fragmented planning. The escalation of armed conflict from 2015 caused widespread damage to infrastructure, displacement of health workers, salary interruptions, and institutional fragmentation, deepening dependence on humanitarian financing and externally driven vertical programmes [14,16]. Subsequent shocks, including recurrent outbreaks and COVID-19, further exposed the limits of the system’s resilience, with emergency responses that were often reactive, short-term, and donor-led.

Despite a growing literature on health system resilience in fragile and conflict-affected settings, empirical evidence from protracted conflict contexts remains limited, and few studies capture the perspectives of national and subnational policymakers across successive phases of crisis [16,17]. This manuscript builds on, but is analytically distinct from, the wider qualitative study reported previously [18]. That publication examined how formal governance persisted, while resilience remained constrained across a decade of conflict and crisis. The present manuscript applies a different policy-architecture lens: it examines how resilience capacities are distributed across the WHO health system building blocks and how decision rights, financing rules, workforce governance, information use, service continuity, and learning mechanisms enable or constrain movement from crisis coping toward transformative institutional capability. Specifically, we ask: (1) which resilience capacities are evident across the building blocks; (2) how do governance, financing, workforce, information, and service-delivery policies enable or constrain these capacities; and (3) what policy conditions would allow crisis coping to accumulate into durable institutional reform. Although grounded in the Yemen case, the analytical framing may inform future testing and adaptation in other conflict-affected settings.

## Methods

### Study design and setting

This article reports a focused qualitative analysis from a wider interview study on health system governance and resilience in Yemen, previously reported in relation to formal governance persistence and system resilience [18]. The present analysis uses the same interview dataset but addresses a distinct question: how strategic policy arrangements across the WHO health system building blocks shaped preparedness, absorption, adaptation, learning, and transformation across successive shocks. We examined four periods: the pre-conflict period before 2014, the active conflict phase from 2015 to 2019, the COVID-19 pandemic, and the current protracted crisis from 2020 to 2025. The analysis focused on the formal health system under the internationally recognised Government of Yemen and the Ministry of Public Health and Population (MoPHP) in Aden, while recognising that health governance in Yemen remains territorially and politically divided. A qualitative design was appropriate because the study sought to examine policymakers’ interpretations of institutional processes, policy decisions, and governance dynamics that are not directly captured through routine quantitative indicators.

### Conceptual framework

The study was guided by two complementary frameworks: the WHO health system building blocks, used to structure inquiry across the major system components, and a resilience maturity spectrum distinguishing preparedness, absorption, adaptation, and transformation [2,19]. The WHO Health Emergency and Disaster Risk Management framework positions emergencies within a continuous cycle of prevention, preparedness, response, and recovery that should be embedded in governance and health policy [20]. Governance was treated as a cross-cutting function operating through authority, coordination, accountability, decision-making, information use, and institutional learning. Preparedness was treated as an enabling precondition for resilience rather than as a capacity equivalent to absorption, adaptation, or transformation, and the capacities were read as coexisting and overlapping rather than as fixed sequential stages [4,17].

### Sampling and participants

Participants were purposively selected to provide senior-level insight into health system governance and crisis response across the study periods. The sample included 30 national and subnational policymakers and health leaders from MoPHP sectors, technical departments, advisory roles related to policy and donor coordination, and academic institutions. Selection was based on institutional role, experience before and after the 2015 escalation of conflict, and involvement in major public health emergencies, including COVID-19. No participant had a supervisory relationship with the research team. Recruitment continued until the interviews produced sufficient depth and recurrence of themes across the main policy domains.

### Data collection

Data were collected through semi-structured, in-depth interviews conducted between May and July 2025 using the same pilot-tested interview guide developed for the wider qualitative study reported previously [18]. The guide is provided as Supplementary Material 1 to allow assessment of the interview domains and their alignment with the present analysis. It covered governance and leadership, health financing and donor coordination, workforce, health information systems, emergency preparedness and response, and institutional learning. Interviews were conducted at participants’ workplaces, lasted 60–120 minutes, and were audio-recorded with consent and supplemented by field notes. Six trained local researchers (one female and five males; master’s students) collected data under the direct supervision of the first author. Interviews were transcribed verbatim in Arabic and anonymised, and selected quotations were translated into English using a meaning-based approach to preserve conceptual integrity.

### Data management and analysis

Data were analysed in ATLAS.ti 25 using framework-informed thematic analysis with combined deductive and inductive coding. The WHO health system building blocks, governance functions, and resilience capacities were used as a priori sensitising categories derived from the study objectives and conceptual framework, while more specific empirical codes were developed inductively from interview transcripts. The study did not aim to generate a formal grounded theory; therefore, we avoid characterising the analysis as a grounded theory. Coding proceeded through repeated reading, line-by-line coding of meaning units, grouping of related codes within and across the a priori domains, and development of themes explaining how policy arrangements shaped preparedness, absorption, adaptation, learning, and transformation. For example, excerpts describing emergency committees, unclear mandates, and post-crisis discontinuity were coded as event-based coordination, fragmented authority, and weak institutionalisation, then grouped under the leadership and governance theme and interpreted as absorptive but weakly transformative capacity. Analysis was iterative and comparative, applying constant comparison within and across interviews and across the four time periods. Analytical memos supported reflexivity and theme refinement. The maturity ratings used in this study (substantial, partial, and limited) are analytic judgements reflecting how consistently each capacity was evidenced across the building blocks and periods, rather than quantitative scores.

### Trustworthiness and rigor

Rigor was strengthened through prolonged engagement with the data, systematic coding, reflexive memo-writing, use of a coding matrix linking a priori domains to inductively developed codes, and temporal comparison across crisis phases. Coding was led by the first author and reviewed by the wider team to support consistency. Reporting followed the Consolidated Criteria for Reporting Qualitative Research (COREQ). A fuller account of the wider study is reported separately [18].

## Results

### Participant characteristics

Thirty senior policymakers and health leaders participated. Most had 20 or more years of professional experience, and the majority were men. Participants were drawn from multiple MoPHP sectors, including planning and international cooperation, primary health care, population and reproductive health, and curative and diagnostic medicine, as well as administrative departments affiliated with the Office of the Minister and academic institutions. Most reflected primarily on the COVID-19 and post-COVID periods, while a smaller group provided perspectives spanning all four phases.

Using the policy-architecture lens developed for this manuscript, the analysis examined how authority, financing, workforce stability, data use, service continuity, and learning mechanisms shaped resilience capacities across the WHO building blocks. The findings below present the same wider qualitative material through a distinct analytical focus: not whether governance persisted during crisis, but whether policy arrangements enabled absorptive and adaptive responses to mature into transformative institutional capability. Across building blocks, the core temporal pattern was weak pre-crisis preparedness, externally supported absorption during conflict and COVID-19, localised and reversible adaptation, and limited movement toward transformation (Table 1; Table 2).

**Table 1.** Resilience maturity spectrum in protracted crisis settings.

Resilience capacity	Governance & leadership	Financing	Health workforce	Health information system	Service delivery	Learning & recovery	Overall maturity assessment
Preparedness	Plans and committees exist but weakly institutionalised; unclear mandates; legal conflict between central and local authorities	No protected emergency reserve; pre-war emergency line not operationalised	No surge roster; hiring freeze since 2011; no structured pre-crisis disaster training	Fragmented, largely manual system pre-COVID; no integrated health information system	No formal continuity of essential services protocol; informal referral pathways	No routine simulation training; limited documentation of preparedness exercises	Low preparedness maturity-formal planning without operational embedding
Absorptive	Rapid activation of crisis committees; ad hoc coordination mechanisms	Donor surge financing; temporary contracting; emergency reallocation	Volunteer contracting; temporary surge staffing; short-term IPC training	eDEWS daily reporting; outbreak alerts; rapid communication loops	Isolation centres, ICU expansion, oxygen supply; partial maintenance of essential services	Crisis response experience accumulated but not systematically captured	Moderate but externally buffered absorptive maturity-collapse prevented, but capacity remained donor-dependent and vulnerable
Adaptive	Crisis coordination structures reused; partial decentralised tailoring; governance fragmentation limits integration	Reallocation during shock; no fiscal redesign; continued donor reliance	Expanded IPC training; uneven competency standards; incentive-driven retention	Establishment of HIS directorates; dashboards and Kobo/GPS use; incomplete interoperability	Repurposing surge assets: PHC outreach maintained; uneven regional adaptation	Partial reflection post-shock but no institutionalised reform pathway	Partial adaptive maturity-technical reconfiguration without system integration
Transformative	Persistent legal fragmentation; weak enforceable accountability; committees not permanently embedded	No protected emergency fund; exchange-rate erosion; wage collapse; no sustainable domestic pooling	Salary collapse; continued hiring freeze; no long-term retention reform	No integrated national command-and-control platform; fragmented data-to-decision loop	Surge assets not fully integrated into financed, regulated service models	No formal After-Action Reviews; donor-led evaluations substitute national learning	Transformative capacity constrained-structural reform blocked by governance and fiscal fragmentation

Note: eDEWS (electronic Disease Early Warning System); HIS (Health Information System); ICU (Intensive Care Unit); IPC (Infection Prevention and Control); Kobo/GPS (Kobo Toolbox/Global Positioning System); PHC Primary Health Care).

**Table 2.** WHO health system building blocks in Yemen: empirical findings and observed resilience capacity.

WHO Building Block	Key Empirical Findings	Dominant Resilience Capacity	Strategic Policy Leverage
Leadership & governance	Fragmented stewardship; legal conflict between central and local authorities; ad hoc crisis committees; weak accountability routines	Absorptive; partial adaptive; transformative constrained	Clarify mandates and decision rights; institutionalise incident management and routine review; embed After-Action Reviews
Financing	Donor-driven flows; weak emergency buffers; COVID asset surge without protected O&M; exchange-rate erosion	Externally buffered absorption; contingent adaptation	Protected emergency financing; safeguard recurrent O and M; align partner funding with national resilience framework
Health workforce	Hiring freeze; salary collapse; reliance on incentives; surge contracting; protection gaps	Fragile absorption; adaptive coping	Stabilise wages; reopen recruitment; institutional surge roster; competency-based supervision
Products & technologies	Oxygen/ICU surge assets; maintenance uncertain; donor-dependent logistics	Improved absorption; partial adaptation	Lifecycle governance (maintenance, utilities, asset registry)
Health information system	Improved outbreak reporting; weak capacity dashboards; limited institutional learning	Absorptive(surveillance); partial adaptation	Integrated health information system; data-to-decision protocols; permanent learning unit
Service delivery	Facility closures; routine care crowd-out; fragmented referral pathways; late quality structures	Uneven absorption; local adaptation	Triggered continuity protocols; referral governance; embed quality and IPC in routine supervision

Note: O and M (Operations and Maintenance).

### Leadership and governance

The central theme in leadership and governance was event-based coordination without institutionalised stewardship. Respondents described a shift from structured but weak stewardship before 2014 to fragmented, personality-driven leadership after 2015. During COVID-19, emergency committees and multisectoral coordination arrangements enabled rapid absorption, but participants indicated that these mechanisms remained event-based and were not institutionalised in routine governance.

A health emergency committee chaired by the Minister meets during outbreaks. When an emergency occurs, stakeholders agree on roles, tasks, and what should be done, but this is for the current emergency situation. (KI-2; Male, 20 years of professional experience)

Governance, therefore, enabled short-term absorption and partial adaptation, but not sustained transformation. Committees could mobilise actors during shocks, yet decision rights, accountability, and learning mechanisms were not consistently retained once the acute phase passed.

### Health financing

The central theme in financing was donor-buffered absorption without protected domestic financial buffers. Before the conflict, an emergency budget line existed but lacked protection and clear trigger rules. After 2015, donor financing increasingly became the effective operating system. COVID-19 funding expanded oxygen, isolation, and emergency capacity, but recurrent operations and maintenance were not secured. Participants consistently described a financing model that helped prevent collapse but reinforced dependency and became vulnerable when external funding declined.

The Ministry of Health depends heavily on external funding because the state budget is limited. Many contracted and volunteer health workers depended on incentives from organisations during emergencies, and when these incentives declined this created a problem. (KI-29; Female, 15 years of professional experience)

Financing was, therefore, reactive rather than anticipatory: resources were mobilised after shocks, while contingency planning, domestic emergency reserves, and alternatives to donor reductions remained weak.

### Health workforce

The central theme in the health workforce was prolonged coping rather than durable adaptation. Workforce resilience was weakened by a hiring freeze since 2011, salary erosion, brain drain, uneven distribution, and reliance on temporary incentives. Emergency contracting and volunteer mobilisation expanded short-term surge capacity during crises, but did not rebuild a stable public workforce. Several participants stressed that workforce constraints were not only numerical but also linked to motivation, rural distribution, and loss of experienced staff.

There is no health system without health workers. Yemen has many excellent health professionals, but many have migrated because of economic, academic, and job conditions. (KI-3; Male, 20 years of professional experience)

These findings suggest that what appeared to be adaptive workforce arrangements were often prolonged coping mechanisms. They maintained services temporarily but did not reconfigure recruitment, remuneration, rural incentives, or workforce governance.

### Medical products and technologies

The central theme in medical products and technologies was emergency asset expansion without lifecycle governance. Surge investments in oxygen supply, isolation centres, intensive care capacity, and ventilators strengthened immediate absorptive capacity during COVID-19. However, respondents indicated that these assets were not consistently embedded in maintenance budgets, staffing plans, utilities, or governance routines. Without lifecycle financing and clear responsibility for upkeep, emergency assets risked becoming temporary surge gains rather than durable system capacity.

During COVID-19, efforts filled an urgent gap: isolation centres were created, oxygen plants were established, intensive care rooms were supported, and ventilators were provided. But if COVID-19 came again, we would face the same problem. (KI-14; Male, 25 years of professional experience)

The gap between emergency procurement and routine maintenance illustrates why absorption does not automatically become transformation. Assets can expand crisis response while remaining vulnerable if not incorporated into recurrent financing and operational planning.

### Health information system

The central theme in the health information system was improved event detection without decision-linked information use. Surveillance reporting improved during COVID-19, but data were not consistently translated into operational decisions. Respondents highlighted weak visibility of intensive care beds, referral coordination, integrated dashboards, and formal after-action reviews. Participants viewed information weaknesses as direct barriers to equity and timely access.

We still do not have information on available intensive care beds. A patient may keep searching for a bed until it is too late; there should be a system that tells us where beds are available and where to refer the patient. (KI-15; Male, 20 years of professional experience)

This indicates that information systems were able to detect events but were less able to guide referral governance, resource allocation, or institutional learning.

### Service delivery

The central theme in service delivery was emergency response expansion with under-protected routine service continuity. Service delivery became geographically and politically fragmented after 2015. During COVID-19, isolation centres and referral workarounds helped prevent complete collapse, but routine services were crowded out in some settings. Participants described trade-offs between emergency response and continuity of essential services.

During the pandemic, many essential services stopped because some health workers were needed inside isolation centres. Some public facilities providing reproductive health services did not stop, and tried to do what they could despite fear and panic.”(KI-5; Female, 25 years of professional experience)

These accounts show that maintaining essential and routine services is analytically distinct from mounting an emergency response. Service continuity requires staffing, referral systems, supply chains, and financing rules that protect non-emergency care during shocks.

Taken together, the building-block themes show that Yemen’s health system progressed unevenly across resilience capacities. Absorption was visible where committees, donor financing, temporary workforce mobilisation, surveillance, surge technologies, and referral workarounds prevented complete collapse. Adaptation was more limited and localised, occurring where actors repurposed structures or resources to sustain services. Transformation remained constrained because these adaptations were not embedded in protected financing, workforce reform, lifecycle asset governance, integrated information systems, referral governance, or formal learning-to-reform mechanisms (Table 2).

## Discussion

Building on the wider qualitative study reported previously [18], this manuscript applied a distinct policy-architecture lens to examine how resilience capacities were distributed across the WHO health system building blocks and whether crisis responses accumulated into transformative institutional capability. This distinction is important because the persistence of formal governance structures does not, by itself, indicate that policy arrangements across financing, workforce, information, service delivery, and learning were aligned for transformation. Across the six building blocks, complete collapse was often averted through externally supported absorption and localised adaptation, but the underlying pattern remained one of procedural preparedness, donor-buffered absorption, reactive and localised adaptation, fragmented learning, and limited transformation. We interpret these findings using resilience capacities as coexisting analytic categories rather than sequential stages, examining how governance arrangements shaped preparedness, absorption, adaptation, learning, and transformation across the building blocks. Preparedness functioned largely as an enabling precondition that was rarely met. Plans and committees existed but were weakly operationalised, without protected financing, pre-positioned resources, or routinely tested protocols, consistent with evidence that preparedness must be embedded as a core governance function with dedicated authority and financing rather than as static documentation [5,8,12]. Absorptive capacity was, therefore, substantial only in an operational and short-term sense: humanitarian financing and donor-led mechanisms helped prevent complete collapse, but this capacity weakened when external funding became less predictable and was not converted into protected domestic buffers, mirroring evidence from other fragile and conflict-affected settings [17,21].

Adaptation was pragmatic but weakly institutionalised. Several responses often labelled as adaptive, such as temporary contracting, incentive-driven retention, repurposed emergency structures, and donor-supported arrangements, are better understood as prolonged coping that sustained absorption without reconfiguring rules or roles; genuine adaptation was more limited and uneven. Learning was the weakest link in this resilience configuration: crisis experience was rarely converted into institutional memory, because after-action reviews, feedback loops, and reform pathways were largely absent, so absorptive and adaptive gains seldom accumulated into transformation [4,18]. Transformative capacity was largely absent; where COVID-19 opened a temporary policy window, reforms were not sustained once the acute phase passed.

Transformative resilience remains constrained by fragmented central-local authority; financing that supports emergency procurement but not recurrent operations and maintenance; workforce instability; outbreak detection without integrated referral governance; and weak mechanisms to translate crisis experience into enforceable reform [2,3]. Equity concerns reinforce the need for system integration: without real-time information on available beds, oxygen supply, or functioning facilities, patients experience delays in accessing care. Capacity dashboards and coordinated referral mechanisms are, therefore, practical equity tools, not only technical improvements.

Strategic health policy, in this setting, requires aligning decision rights, financing rules, workforce governance, regulatory standards, information systems, and accountability routines so that crisis coping becomes sustained capability. Transformation does not require wholesale redesign. It requires institutionalising durable maturity markers: clarified authority, audited emergency financing with protected operations and maintenance, regulated surge workforce mechanisms, lifecycle governance of technologies, integrated health information systems linked to action, and formal learning to implementation processes [2,4].

Without these institutional anchors, resilience remains reactive; with them, absorptive and adaptive gains can accumulate into transformation.

### Strengths and limitations

The maturity ratings used here (substantial, partial, and limited) are analytic judgements derived from the resilience maturity spectrum, reflecting how consistently each capacity was evidenced across the six building blocks and the four time periods, rather than quantitative scores. Several limitations should be noted. The analysis draws on retrospective accounts from senior policymakers, which may be subject to recall and social desirability bias, particularly on politically sensitive questions of authority and accountability. Because the findings reflect the perspectives of national and subnational decision-makers within the internationally recognised health authorities, they capture policymaker perceptions rather than a system-wide audit and may not represent front-line, community, or non-state viewpoints. Conducting interviews in an active conflict setting also constrained access and may have introduced selection bias, and Yemen’s fragmented governance means some accounts reflect localised rather than nationally uniform experience.

## Conclusion

Yemen demonstrates that externally supported absorptive resilience and partial local adaptation can prevent complete system collapse under prolonged conflict and repeated shocks. Essential services were sustained through surge assets, temporary workforce mobilisation, intensified surveillance, referral workarounds, and donor-supported financing. However, these responses were crisis-driven, donor-dependent, and unevenly institutionalised, and, therefore, did not amount to durable transformative resilience. Transformative resilience remains constrained by fragmented authority between central and local levels; unprotected operations and maintenance financing; workforce instability; an information system that detects outbreaks without enabling referral governance; and weak institutional learning.

In protracted crisis settings, resilience becomes durable only when crisis coping is converted into institutional capability. Progress should, therefore, be judged not only by the survival of the next shock, but by whether each crisis leaves the system more coherent and more equitable than before.

## Supplementary Material

Supplementary_material_1_clean.docx

COREQ_Checklist updated 22 Aug.pdf

## Data Availability

Data are available from the corresponding author upon reasonable request.

## References

[1] WHO. Everybody’s business: strengthening health systems to improve health outcomes: WHO’s framework for action. Geneva: World Health Organization; 2007 [cited 2026 Jul 21]. Available from: https://www.who.int/publications/i/item/everybody-s-business--strengthening-health-systems-to-improve-health-outcomes

[2] Kruk ME, Myers M, Varpilah ST, et al. What is a resilient health system? Lessons from ebola. Lancet. 2015;385:1910–9. doi: 10.1016/S0140-6736(15)60755-3 25987159

[3] Blanchet K, Nam SL, Ramalingam B, et al. Governance and capacity to manage resilience of health systems: towards a new conceptual framework. Int J Health Policy Manag. 2017;6:431. doi: 10.15171/ijhpm.2017.36 PMC5553211 28812842

[4] Hanefeld J, Mayhew S, Legido-Quigley H, et al. Towards an understanding of resilience: responding to health systems shocks. Health Policy Plan. 2018;33:355–367. doi: 10.1093/heapol/czx183 PMC6277919 29325025

[5] Saulnier DD, Blanchet K, Canila C, et al. A health systems resilience research agenda: moving from concept to practice. BMJ Glob Health. 2021;6:e006779. doi: 10.1136/bmjgh-2021-006779 PMC8344286 34353820

[6] Belloni G, Monod S, Poroes C, et al. Health systems governance, shocks and resilience: a scoping review of key concepts and theories. BMJ Glob Health. 2025;10:e017358. doi: 10.1136/bmjgh-2024-017358 PMC12181996 40541279

[7] Copeland S, Hinrichs-Krapels S, Fecondo F, et al. A resilience view on health system resilience: a scoping review of empirical studies and reviews. BMC Health Serv Res. 2023;23:1297. doi: 10.1186/s12913-023-10022-8 PMC10675888 38001460

[8] WHO. Building health system resilience to public health challenges: guidance for implementation in countries. Geneva: World Health Organization; 2024 [cited 2026 Jul 21]. Available from: https://www.who.int/publications/i/item/9789240094321

[9] United Nations Office for the Coordination of Humanitarian Affairs. Yemen humanitarian needs and response plan 2025. NY/Geneva: United Nations; 2025 [cited 2026 Jul 21]. Available from: https://yemen.un.org/en/287505-yemen-humanitarian-needs-and-response-plan-2025-january-2025

[10] WHO. HeRAMS Yemen baseline report 2023: operational status of the health system. Geneva: World Health Organization; 2023 [cited 2026 Jul 21]. Available from: https://www.who.int/publications/m/item/herams-yemen-baseline-report-2023-operational-status-of-the-health-system

[11] WHO. HeRAMS Yemen summary update report February 2024. Geneva: World Health Organization; 2024 [cited 2026 Jul 21]. Available from: https://www.who.int/publications/m/item/herams-yemen-summary-update-report-2024-02

[12] Garber K, Fox C, Abdalla M, et al. Estimating access to health care in Yemen, a complex humanitarian emergency setting: a descriptive applied geospatial analysis. Lancet Glob Health. 2020;8:e1435–e43. 10.1016/S2214-109X(20)30359-4 PMC7561303 33069304

[13] Kotiso M, Qirbi N, Al-Shabi K, et al. Impact of the COVID-19 pandemic on the utilisation of health services at public hospitals in Yemen: a retrospective comparative study. BMJ Open. 2022;12:e047868. doi: 10.1136/bmjopen-2020-047868 PMC8724586 34980605

[14] Dureab F, Hussain T, Sheikh R, et al. Forms of health system fragmentation during conflict: the case of Yemen. Front Public Health. 2021;9:659980. doi: 10.3389/fpubh.2021.659980 PMC8311287 34322468

[15] Qirbi N, Ismail SA. Health system functionality in a low-income country in the midst of conflict: the case of Yemen. Health Policy Plan. 2017;32:911–922. doi: 10.1093/heapol/czx031 28402469

[16] Martineau T, McPake B, Theobald S, et al. Leaving no one behind: lessons on rebuilding health systems in conflict- and crisis-affected states. BMJ Glob Health. 2017;2:e000327. doi: 10.1136/bmjgh-2017-000327 PMC5656126 29082000

[17] Truppa C, Yaacoub S, Valente M, et al. Health systems resilience in fragile and conflict-affected settings: a systematic scoping review. Confl Health. 2024;18:2. doi: 10.1186/s13031-023-00560-7 PMC10763433 38172918

[18] Hyzam D, Boah M, Dhaiban M, et al. When formal governance persists but resilience does not: health system governance and resilience in Yemen across a decade of conflict and crisis (2014–2025). BMC Health Serv Res. 2026;26:851. 10.1186/s12913-026-14957-6 PMC13282849 42316231

[19] Gilson L, Barasa E, Nxumalo N, et al. Everyday resilience in district health systems: emerging insights from the front lines in Kenya and South Africa. BMJ Glob Health. 2017;2. doi: 10.1136/bmjgh-2016-000224 PMC5656138 29081995

[20] WHO. Health emergency and disaster risk management framework. Geneva: World Health Organization; 2019 [cited 2026 Jul 21]. Available from: https://www.who.int/publications/i/item/9789241516181

[21] Bertone MP, Jowett M, Dale E, et al. Health financing in fragile and conflict-affected settings: what do we know, seven years on? Soc Sci Med. 2019;232:209–219. doi: 10.1016/j.socscimed.2019.04.019 31102931

