# Supplementary material for: Strategic health policy and health system resilience in protracted crisis settings: policymaker perspectives from Yemen
Source: Glob Health Action. 2026 Sep 25;19(1):2734009. doi: 10.1080/16549716.2026.2734009 (PMC13618199; doi:10.1080/16549716.2026.2734009)
Supplement: Supplementary file 2 [file ZGHA_A_2734009_SM8617.docx]

**Health System Resilience: Responding to the Changing Contexts of Health System Challenges and their Impact on the Continuity of Essential Health Services in Yemen**

**Interview Guide for Policy-makers**

This project aims to assess health system performance before crises (2014), evaluate health system resilience through its responses during conflict and COVID-19 and assess its impact on the continuity of essential health services. Additionally, to measure the readiness of public health facilities against future shocks by identifying the gap between the current and required levels of health service needs in real-time, which will determine the dimensions that enhance or inhibit achieving SDG 3 in Yemen.

This tool is conducted to identify the performance of health system building blocks (leadership and governance, Finance, Service delivery, workforce, Essential medicine and technologies, health information) before the stress such as conflict, and COVID-19 pandemic. Additionally to investigate health system resilience capacity through its responses (leadership and governance, Finance, Service delivery, workforce, Essential medicine and technologies, health information) during the emergencies such as conflict and COVID-19 pandemic.

|  | Serial number of interview |
| --- | --- |
|  | Name of institution |
|  | Name of Respondent |
| ______Male, _______Female | Gender |
|  | Academic and professional background |
|  | Current role/position |
|  | Period of working in your position in this institution |
|  | Experience having in total working |
|  | Date of Interview |
|  | Interview time start |
|  | Interview time end |
|  | Name of Interviewer and Signature |
|  | Name of note taker and Signature |

**Informed Consent**

***Note to interviewer: Please read this paragraph to participants prior to the start of the interview. Ensure that participants understand what was read before asking for consent.***

My name is _________________________, and I work as a researcher in this study, which is conducted by Akkon University of Human Science/IRIA - Berlin-Germany. The study attempts to assess the health system resilience capacity in Yemen. This study will be conducted in five governorates: Aden, Taiz, Hadramot, Abyan, and Lahj.

This research will provide the Government of Yemen, represented by the Ministry of Public Health and Population (MoPHP), with the information needed to gain in-depth insight into Yemen's health system resilience capacity.

We would like to ask you some questions to understand more about the situation of health system performance before 2014 and during crises such as conflict and COVID-19. The interview is expected to last 45 minutes to one hour. We may audio-record the interview to help ensure we capture all the critical information from this discussion.

There are no right or wrong answers, and you can refuse to answer any question and terminate this interview at any time. Please note that any responses you give during this interview will be confidential. While your information may be used in our reports, your name or identifying information will not be included. Finally, if your feedback is used in the report, it is crucial to understand that this does not mean the issues raised will lead to immediate changes in the future.

| Consent | | |
| --- | --- | --- |
| ☐ **No**  Terminate interview | ☐ **Yes**  Continue with interview questions | Do you agree to participate in this interview? |
| ☐ **No**  Do not record | ☐ **Yes**  Start recording | Do you agree to have this interview audio-recorded? |
| If no, please write the interviewee's responses. | | |

| **Part 1: Health system performance before 2014 and responding during shocks**  1- Can you briefly describe your role and how it relates to health system governance and policy implementation? | | | | |
| --- | --- | --- | --- | --- |
| **Health system building blocks** | | **Stages** | | |
| **1.Governance** | **Sub-Title** | **1.Health system performance before 2014** | **2.Health System Resilience 2015-2019 (During civil war/conflict /political crisis and its impacts(** | **3.Health System Resilience 2020 (During COVID-19)** |
|  | **1.1.Effective and Participatory Leadership with Strong Vision and Communication** | **1.1.1.1** What is effective leadership from your point of view?  **1.1.1.2** Can you provide me names of effective leaders or at least period you believe the leadership was effective before 2014, such as departments, sectors or even GHOs?  **1.1.1.3** How did effective leadership and decision-making contribute to in **strengthening Yemen's health system function and resilience preparedness capacity before 2014?** Can you provide specific examples of contingency plans, protocols, or emergency legislation developed during this period? How were the practical mechanisms? ***Probe*** Can you give me some examples, references, articles, and experiences? ***Probe***  **1.1.1.4** Was the leadership at that time having a clear vision of the health system? How was the MoPHP making decisions at that time? Was there any participatory mechanisms, including consultants in MoPH and were GHOs involved? Was it a bottom-up approach or the opposite? ***Probe***  **1.1.1.5** How did the health system **prevent, detect and address** public health threats **before 2014?** In terms of stakeholder involvement), stakeholder participation in decision making, engagement, clear and feasible plans for response measures, and quality strategic direction? ***Probe***  **1.1.1.6** What challenges did leaders face, and how were they addressed in terms of contingency planning and protocol, emergency legislation, and stakeholder coordination? Were they included in the health strategies of Yemen then? How was the mechanism? ***Probe*** Can you give me some examples/references/articles/experiences? ***Probe*** | **1.1.2.1** Can you provide me names of effective leaders or at least period you believe the leadership was effective during war/Conflict, such as departments sectors or even GHOs?  **1.1.2.2.** How was leadership **during and after conflict**? At the MoPHP and GHOs levels?  **1.1.2.3** How was the MoPHP making decisions at that time? Was there any participatory mechanisms, including consultants in MoPH and were GHOs involved? Was it a bottom-up approach or the opposite? Have they continued with the same vision? If not when this was developed then? ***Probe***  **1.1.2.4**. How did the health system **prevent, detect and address** public health threats **before** and **during conflict**? In terms of stakeholder participation and engagement, a clear and feasible plan for response measures, setting strategic direction, leadership/steering and a clear chain of command, established public trust in response agencies, and effective communication. Can you give me more details? ***Probe***  **1.1.2.5** What challenges did leaders face, and how were they addressed in terms of contingency planning and protocol, emergency legislation, and stakeholder coordination? Were they included in the health strategies of Yemen then? How was the mechanism? ***Probe*** Can you give me some examples/references/articles/experiences? ***Probe***  **1.1.2.6** Can you explain how the consequences of the civil war/conflict/political crisis mentioned below affected health system function, structure, and indicators? ***Probe***  **1. Economic Shocks: Currency Devaluation-2014**  **2. Humanitarian Crises: Food Insecurity - ongoing since 2014**  **3. Cholera Outbreak (2016-2019)**  **4. Trade Restrictions (2014-Present)**  **5-Displacement and Migration (Ongoing since 2014)**  **6-Environmental shock Drought (2014-2020) leads to increased food insecurity and health challenges.**  **1.1.2.7** How did the MoPHP prepare for and respond to the negative impact of the consequences of war/conflict mentioned above? ***Probe***  Were there strategies to address the effects of the consequences of the war, specifically climate change, on the most vulnerable populations? What role did the ministry play in mitigating the environmental shock? ***Probe*** | **1.1.3.1** Can you provide me names of effective leaders or at least period you believe the leadership was effective during COVID-19, such as departments, sectors or even GHOs?  **1.1.3.2** How was leadership **before and during COVID-19**? At the MoPHP and GHOs levels?  **1.1.3.3** How was the MoPHP making decisions at that time? Was there any participatory mechanisms, including consultants in MoPH and were GHOs involved? Was it a bottom-up approach or the opposite?Have they continued with the same vision? If not when this was developed then? ***Probe***  **1.1.3.4** How did the health system **prevent, detect and address** public health threats **before and during COVID-19**? Can you describe specific actions, such as risk communication, stakeholder coordination, or adaptive policies, a clear and feasible plan for response measures, setting strategic direction, leadership/steering and a clear chain of command, established public trust in response agencies, and effective communication that were implemented to benefit society? Can you give me more details? ***Probe***  **1.2.3.5** What challenges did leaders face, and how were they addressed in terms of contingency planning, emergency legislation, and stakeholder coordination?" Were they included in the health strategies of Yemen then? How was the mechanism? ***Probe*** Can you give me some examples/references/articles/experiences? ***Probe***  **1.1.3.6** Can you explain **how** COVID-19 affected health system function; structure; and indicators? ***Probe***  **1.1.3.7** How did the MoPHP respond and manage the impacts of COVID-19 on health indicators? |
|  | **2.Coordination of Activities across Government and Key Stakeholders** | **1.2.1.**1 What can you tell me about the coordination of activities across government and key stakeholders **before 2014?**  **1.2.1.2** Was there any coordination of activities across different sectors and levels of government to strengthen **health system function and resilience preparedness capacity before 2014**? Which sectors? What were the inputs and outputs of multi-sector coordination? ***Probe***  **1.2.1.3.** What agreements were established with relevant actors, such as private sector, international agencies and NGOs, to enhance coordinated action? Can you give me some examples? ***Probe*** | **1.2.2.**1 What can you tell me about the coordination of activities across government and key stakeholders **during conflict?**  **1.2.2.2** Was there any coordination of activities across different sectors and levels of government **during and after conflict and its consequences**? Which sectors?  **1.2.2.3** How did the coordination of activities across different sectors and levels of government contribute to effective collaboration **during and after conflict and its consequences**? ***Probe***  **1.2.2.4** What agreements were established with relevant actors, such as private sector, international agencies and NGOs, to enhance coordinated action during conflict and its impacts? Can you give me an example? Furthermore, how is its impact reflected on health system responses during the crisis and its consequences? ***Probe*** | **1.2.3.**1 What can you tell me about the coordination of activities across government and key stakeholders **during COVID-19?**  **1.2.3.2** Was there any coordination of activities across different sectors and levels of government **Pre- and** **during COVID-19**? Which sectors?  **1.2.3.3** How did coordinating activities across different sectors and levels of government contribute to effective collaboration **Pre- and** **during COVID-19**? ***Probe***  **1.2.3.4.** What agreements were established with relevant actors, such as private sector, international agencies and NGOs, to enhance coordinated action? Can you give me an example? Moreover, how is its impact reflected on the health system responses **Pre- and** **during COVID-19**? ***Probe*** |
|  |  |  |  |  |
|  | **3-Organizational Learning Culture that is Responsive to Crises** |  | **1.3.2.1** What can you tell me about the culture of learning during crises? ***Probe***  **1.3.2.**2 How did the organizational learning culture contribute for adaptation **during conflict**?? ***Probe***  **1.3.2.3.** What mechanisms were implemented to assess and learn from the response to the crisis, and how did they inform future decision-making?  **1.3.2.4** What were the lessons learned **after war and during conflict** in terms of contingency planning, emergency legislation, and stakeholder coordination? ***Probe*** | **1.3.3.1** How did the organizational learning culture contribute **after COVID-19**? ***Probe***  **1.3.3.2** What mechanisms were in place to assess and learn from the response to COVID-19, and how did they inform future decision-making?    **1.3.3.3** What were the lessons learned **after COVID-19** in terms of contingency planning, emergency legislation, and stakeholder coordination? ***Probe*** |
|  | **4-Effective Information Systems and Flows** | **1.4.1.1 How was the functionality of HIS before 2014?** **What kind of HIS were available before 2014?**  **1.4.1.2.** How did health information systems support decision-making by ensuring the timely sharing of critical information with stakeholders **before 2014**? ***Probe***  **1.4.1.3** What communication infrastructure was in place to facilitate the flow of information and data-sharing among key decision-makers **before 2014**? ***Probe***  What technologies or platforms were used for sharing information? provide specific case studies or examples of effective information-sharing practices  **1.4.1.4** Were there specific protocols for ensuring data accuracy and reliability **before 2014**?  **1.4.1.5** How were marginalized or remote communities included in the information-sharing process **before 2014**? | **1.4.2.1 How was the functionality of HIS during –war/ conflict? What kind of health information system were available during conflict?**  **1.4.2.2** How did health information systems support decision-making by ensuring the timely sharing of critical information with stakeholders **during conflict and the abovementioned crisis**? ***Probe***  **1.4.2.3** What communication infrastructure was in place to facilitate the flow of information and data-sharing among key decision-makers **during conflict and its consequences mentioned above**? ***Probe***  What technologies or platforms were used for sharing information? provide specific case studies or examples of effective information-sharing practices  **1.4.2.4** Were there specific protocols for ensuring data accuracy and reliability **during conflict**?  **1.4.2.5** How were marginalized or remote communities included in the information-sharing process **during conflict**?  **1.4.2.6** What role did technology (e.g., telemedicine, digital health tools, symposia. etc) play in maintaining service delivery **during conflict?** | **1.4.3.1 How was the functionality of HIS during COID-19 and in the real-time? What kind of health information system were available during and after COVID?**  **1.4.3.2** How did health information systems support decision-making by ensuring timely sharing of critical information with stakeholders **Pre- and during COVID-19**? ***Probe***  **1.4.3.3** What communication infrastructure was in place to facilitate the flow of information and data-sharing among key decision-makers **Pre- and during COVID-19**? ***Probe***  What technologies or platforms were used for sharing information? provide specific case studies or examples of effective information-sharing practices  **1.4.3.4** Were there specific protocols for ensuring data accuracy and reliability **during COVID-19**?  **1.4.3.5** How were marginalized or remote communities included in the information-sharing process during **COVID-19**?  **1.4.3.6** What role did technology (e.g., telemedicine, digital health tools, symposia. etc) play in maintaining service delivery **during COVID-19?**  **1.4.3.7**. How did the health system tailor public health messaging for patients about the risk of COVID-19? **For example: community involvement**. ***Probe***  1.4.3.8 What positive changes have been made to health information systems as lessons learned from the COVID-19 pandemic? ***Probe*** |
|  | **5-Surveillance Enabling Timely Detection of Shocks and Their Impact** | **1.5.1.1** How do you evaluate Yemen's epidemiological surveillance and early warning systems **before 2014**? ***Probe***  **1.5.1.2.** Were there any mechanisms to identify change in need and access to services in health system strategies **before 2014**? ***Probe*** | **1.5.2.1** How did surveillance systems detect and track events in real-time during the crisis? ***Probe***  **1.5.2.2**. What mechanisms existed to identify changes in needs and access to services, and how did they contribute to practical response efforts during the abovementioned crisis? ***Probe*** | **1.5.3.1** How do you evaluate the epidemiological surveillance and early warning systems in Yemen **Pre- and during COVID-19**? ***Probe***  **1.5.3.2** Were there any mechanisms to identify change in need and access to services **during COVID-19**? ***Probe***  1.5.3.3 What are the lessons learned for developing epidemiological surveillance and early warning systems after the pandemic? If not, why? ***Probe*** |
| **2-Financing** | **6-Ensuring Sufficient Monetary Resources in the System and Flexibility to Reallocate and Inject Extra Funds** | **2.6.1.1What were the funding mechanisms available? And how were health system functions funded before 2014?**  **2.6.1.2** Was the health system adequately funded to respond effectively to delivering health services as a routine health system operation? Can you estimate the expenditure on health (GDP, government spending on health, out-of-pocket) **before 2014 in Yemen**? ***Probe***  **2.6.1.3** What measures were taken to inject extra funds into the health system strategies to maintain the delivery of essential health services as a preparedness capacity against future shock **before 2014 in Yemen**? **In term of monetary resources in the system and flexibility to reallocate and inject extra funds, Purchasing flexibility and reallocation of funding to meet changing needs**  ***Probe*** | **2.6.2.1 What were the funding mechanisms available? And how was health system functions funded during War? *Probe***  **2.6.2.2** What measures were taken to inject extra funds into the health system to maintain essential services during war/conflict and its impacts? **In term of monetary resources in the system and flexibility to reallocate and inject extra funds, Purchasing flexibility and reallocation of funding to meet changing needs. *Probe*** | **2.6.3.1 What were the funding mechanisms available? And how was health system functions funded during COVID-19? And in real-time?**  **2.6.3.2** What measures were taken to inject extra funds into the health system and to maintain the delivery of essential health services in a preparedness capacity **during COVID-19 and in real time**? **In term of monetary resources in the system and flexibility to reallocate and inject extra funds, Purchasing flexibility and reallocation of funding to meet changing needs. *Probe*** |
|  | **7- Comprehensive Health Coverage** | **2.7.1.1** How was the essential health services planning contributing to better health services coverage for population **before 2014**? ***Probe***  **2.7.1.2** What type of health financing available **before 2014** (participatory, taxation or both)? Was the Universal health coverage part of it? What about the out of pocket? ***Probe***  **2.7.1.3** Did the health system adopt in its strategies an evidence-based package of services to contribute to the resilience of healthcare activities against future shocks **before 2014**? ***Probe*** | **2.7.2.1** What gaps were identified in health coverage, and which method did you use to identify these gaps? Moreover, which mechanisms did you adopt in health system strategies during **conflict** to avoid exacerbating the crisis, particularly for (vulnerable groups, services are not covered, out-of-pocket payments)? ***Probe*** | **2.7.3.1** What gaps were identified in health coverage, which method did you use to identify them, and which mechanisms did you adopt to avoid exacerbating the crisis, particularly for (vulnerable groups, services are not covered, out-of-pocket payments) **during COVID-19 and in real-time**? ***Probe*** |
|  | **8-Ensuring Stability of Health System Funding through Countercyclical Health Financing Mechanisms and Reserves** |  | **2.8.2.1** What mechanisms were in place to prevent health services funding from collapse **during conflict**?  **2.8.2.2** Were there countercyclical financing mechanisms that helped cushion the financial impact of economic shock on the health system **during war and conflict**? ***Probe***  **2.8.2.3** What financial reserves were available for deployment **during conflict, and what were their impacts**? ***Probe*** | **2.8.3.1** What financial reserves were available for deployment **during COVID-19**? ***Probe***  2.8.3.2 Are there any measures taken after the pandemic to avoid any future financing-related crises as a lesson learned? If yes, what are they? If no, why? ***Probe*** |
|  | **9-Purchasing Flexibility and Reallocation of Funding to Meet Changing Needs** |  |  | **2.9.3.1** How did the health system adapt its purchasing mechanisms to address shifts in demand for certain types of Care (Supply chain) **during COVID-19**? ***Probe***  **2.9.3.2** What strategies were adopted to reallocate funding to different providers or activities in response to changing needs during COVID-19? ***Probe***  2.8.3.3 Were there any measures taken after the pandemic to avoid any future crisis related to procurement mechanisms as a lesson learned? If yes, what were they? If no, why? ***Probe*** |
| **3-Resource generation** | **10-Appropriate Level and Distribution of Human and Physical Resources** | **3.10.1.1** How did the health system ensure that human and physical resources were sufficient and appropriately distributed **before 2014**? ***Probe***  **3.10.1.2**. What measures were taken to assess the capacity of diagnostics, primary, and specialist care as a routine health system operation **before 2014**? ***Probe***  **3.10.1.3** How did the MoPHP manage the availability of pharmaceuticals, medical products, vaccines, and equipment **before 2014**? ***Probe*** | **3.10.2.1**. How did the health system ensure that human and physical resources were sufficient and appropriately distributed **during conflict and its consequences**? And how did the health system address workforce shortages and capacity gaps during crises? ***Probe***  **3.10.2.2** What measures were taken to assess the capacity of diagnostics, primary, and specialist care **during conflict and its consequences**? ***Probe***  **3.10.2.3** How did the MoPHP manage the availability of pharmaceuticals, medical products, vaccines, and equipment **during conflict and its consequences**? ***Probe***  **3.10.2.4** How did the MoPHP manage the mapping of health service providers (location, type, opening hours, and accessibility) **during conflict and its consequences**? ***Probe***  **3.10.2.5** How did the MoPHP manage the numbers of doctors and nurses and their workload **during conflict and its consequences**? ***Probe***  **3.10.2.6**. How did the MoPHP manage Workforce mapping (location, availability, competencies) **during conflict and its consequences**? ***Probe*** | **3.10.3.1**. How did the health system ensure that human and physical resources were sufficient and appropriately distributed **before/during COVID-19 and in the real- time**? And how did the health system address workforce shortages and capacity gaps during crises? ***Probe***  **3.10.3.2** What measures were taken to assess the capacity of diagnostics, primary, and specialist care **Pre-/ during COVID-19 and in real-time**? ***Probe***  **3.10.3.3.** How did the MoPHP manage the availability of pharmaceuticals, medical products, vaccines, and equipment **during COVID-19**? ***Probe***  **3.10.3.4.** How did the MoPHP manage the mapping of health service providers (location, type, opening hours, and accessibility) **during COVID-19**? ***Probe***  **3.10.3.5**. How did the MoPHP manage the numbers of doctors and nurses and their workload **during COVID-19**? ***Probe***  **3.10.3.6**. How did the MoPHP manage Workforce mapping (location, availability, competencies) **during COVID-19**? ***Probe***  **3.10.3.7** Were there any measures taken after the pandemic to avoid any future crises regarding the distribution of labor and non-human resources as a lesson learned? If yes, what were they? If no, why? ***Probe*** |
|  | **11-Ability to increase capacity to cope with a sudden surge in demand** |  | **3.11.2.1** How was the health system's ability to increase the number of health professionals, their workload, and workforce reserves **during conflict and its consequences**? ***Probe***  **3.11.2.2**. How did MoPHP increase service capacity (e.g., waiting lists and occupancy rates) **during conflict and its consequences**? ***Probe***  **3.11.2.3** Was there an agency responsible for emergency supplies **during conflict and its consequences**? ***Probe*** | **3.11.3.1** How was the health system's ability to increase the number of health professionals, their workload, and workforce reserves **during COVID-19**? ***Probe***  **3.11.3.2** How was the ability of MoPHP to increase the capacity of services (e.g., existence of waiting lists, occupancy rates) **during COVID-19**? ***Probe***  **3.11.3.3** How was the ability of MoPHP to increase the number of health professionals, their workload, and workforce reserves **during COVID-19**? ***Probe***  **3.11.3.4** Was there an agency responsible for emergency supplies d**uring COVID-19**? ***Probe*** |
|  | **12-Motivated and well-supported workforce** | **3.12.1.1** What strategies were in place to address the mental health needs of healthcare workers and the general population during crises? | **3.12.2.1** Were there specific programs to provide psychosocial support in conflict-affected areas? How effective were they? How did the health system ensure continuity of mental health services **during the crisis**?  **3.12.2.2** What mechanisms used to motivate HWF during crises (**conflict**)?  **3.12.2.3** How was the health system's ability to ensure health workers' safety **during conflict and its consequences**? ***Probe***  **3.12.2.4** How did the MoPHP ensure staff support mechanisms helplines **during conflict and its consequences**? ***Probe*** | **3.12.3.1** Were there specific programs to provide psychosocial support during the **COVID-19 pandemic**? How effective were they? How did the health system ensure continuity of mental health services during that time? What about in **real-time**?  **3.12.3.2** What mechanisms used to motivate HWF during **COVID-19**?  **3.12.3.3** How was the health system's ability to ensure health workers' safety **during COVID-19**? ***Probe***  **3.12.3.4** How did the MoPHP ensure staff support mechanisms and helplines **during COVID-19**? ***Probe***  **3.12.3.5** Have any measures been taken post-pandemic to address the mental health needs of healthcare workers and the general population? If yes, what are they? If no, why? ***Probe*** |
| **4-Service delivery** | **13-Alternative and Flexible Approaches to Deliver Care** | **4.13.1.1** How did the health system adapt its essential healthcare service package delivery to meet health care quality and safety standards across all services as a routine health system operation, and how was it planned **before 2014**? ***Probe***  **4.13.1.2** What alternative pathways for care delivery were established in the health system strategies **before 2014** to ensure service continuity delivering in the face of disruptions? ***Probe***  **4.13.1.3** Was the crisis preparedness training cross-training for additional skills a part of health strategies **before 2014**? Was the trading program continuously provided? Can you give me some examples based on the general topics of training program? ***Probe***  **4.13.1.4.** Were there training programs for health workers to treat specific or at-risk population groups **before 2014**? ***Probe*** | **4.13.2.1** What alternative pathways for care delivery were established by the health system **during conflict and its consequences** to ensure service continuity in the face of disruptions? ***Probe***  **What about Minimal health service package?**  **4.13.2.2**. Was the crisis preparedness training and cross-training for additional skills a part of the health system risk management plan helplines **during and after conflict and its consequences**? ***Probe***  **4.13.2.3** What training programs were implemented to build resilience capacity among health workers and policymakers?  **4.13.2.4** Was there training of health workers to treat specific or at-risk population groups **during conflict and its consequences**? ***Probe***  **4.13.2.5** How did the MoPHP ensure the provision of services for at-risk population groups **during conflict and its consequences**? ***Probe***  **4.13.2.6** How did the MoPHp maintain quality and safety standards across all services **during the conflict and its consequences**? ***Probe*** | **4.13.3.1** How were the adaptive policy for health services delivery **during COVID-19**? ***Probe***  **Examples*:* 1. Telemedicine, 2. Restructure the health care provider system, 3-Reduction of supportive treatments, 4-Prioritisation and triage**  **4.13.3.2.** What alternative pathways for care delivery were established in the health system strategies **Pre-and during COVID-19** to ensure service continuity in the face of disruptions? ***Probe***  **4.13.3.3** Was the crisis preparedness training cross-training for additional skills a part of the health system risk management plan **Pre - and during COVID-19**? ***Probe***  **4.13.2.4** What training programs were implemented to build resilience capacity among health workers and policymakers?  **4.13.3.5** Was there training of health workers to treat specific or at-risk population groups **Pre - and during COVID-19**? ***Probe***  **4.13.3.6** How did the MoPHP ensure the provision of services for at-risk population groups **during COVID-19**? ***Probe***  **4.13.3.7** How did the MoPHO maintain quality and safety standards across all services **during COVID-19**? ***Probe***  **4.13.3.8** Were there initiatives to strengthen institutional capacity for crisis preparedness and response regarding providing pf essential health services before and after COVID-19? ***Probe*** |

**Part 2: Health system resilience assessment**

1- How does the MoPHP of Yemen currently define health system resilience? How has this definition evolved?

2- In Yemen, does the government or the MoPHP assess the resilience capacity of the health system?

**If Yes: When/What?**

**If No: Why?**

**If Ongoing: Can you give some examples?**

**2.2 If you answered YES to Q2:**

| **2.2.1 What health system sub-parts are covered by the assessment?** | **covered by the assessment** | **Needs the assessment** |
| --- | --- | --- |
| 1-Public health |  |  |
| 2-Primary Care |  |  |
| 3-Emergency Care |  |  |
| 4-Hospital Care |  |  |
| 5-Outpatient specialist care |  |  |
| 6-Long-term Care (chronic diseases) |  |  |
| 7-Care coordination (cross-sectoral) |  |  |
| 8-others |  |  |

| **2.2.2- What are the key elements covered by the assessment?** | **covered by the assessment** | **Needs the assessment** |
| --- | --- | --- |
| 1-Human resources |  |  |
| 2-Pharmaceuticals and medical supplies/equipment |  |  |
| 3-Health information management |  |  |
| 4-Financing / contingency funding |  |  |
| 5-Crisis preparedness plan(s) |  |  |
| 6-others |  |  |

**3-Which specific capacities for health system resilience are covered by the assessment? Probe**

**▢ Preventive/forecasting capacity**, i.e., the ability of the health system to proactively foresee the advent of a shock and minimize its potential future impact. **Examples**: (covered by the assessment/Needs the assessment)

**1. Epidemic Surveillance Systems**

**2. Vaccination Programs**

**3. Health Risk Assessments**

**4. Epidemiological Training programs**

**5. Public Health Education Campaigns**

**6. Collaboration with Research Institutions**

**7. Data Analytics and Modeling**

**8. Community Engagement and Feedback Mechanisms**

**▢ Absorptive capacity**, i.e., Absorptive capacity refers to the ability of a health system to withstand shocks and maintain essential functions during a crisis. It involves the capacity to absorb the impact of a shock without significant disruption to services. **Examples**: (covered by the assessment/Needs the assessment)

**1. Emergency Response Protocols**

**2. Surge Capacity Planning**

**3. Stockpiling Essential Supplies**

**4. Flexible Workforce Management**

**5. Community Health Programs**

**6. Telehealth Services**

**7. Partnerships and Collaborations**

**8. Mental Health Support Services**

**9. Data-Driven Decision Making**

**▢ Adaptive capacity**, i.e., Adaptive capacity refers to the ability of a health system to adjust its operations and strategies in response to changing circumstances and emerging challenges during a crisis. **Examples**: (covered by the assessment/Needs the assessment)

**1. Pandemic Response**

**2. Resource Shortages**

**3. Health Workforce Management**

**4. Community Engagement**

**5. Data Utilization**

**▢ Transformative capacity,** i.e., Transformative capacity refers to the ability of a health system to fundamentally change its structure, processes, and practices in response to a crisis, leading to long-term improvements and resilience.

**Transformative Capacity of the Health System: Governance and Leadership**

1. Has a national health risk management authority been established to operate permanently and manage crises in a coordinated manner? When? If not, why not?

2. Are communities engaged in decision-making to enhance transparency and accountability? When? If not, why not?

3. Is collaborative multisectoral governance being implemented? When? If not, why not?

4. Has a nationwide electronic system been developed to collect real-time data? When? If not, why not?

**Transformative Capacity of the Health System: Health Financing**

1. Has a national health emergency fund been established? When? If not, why not?

2. Has there been a gradual transition toward a universal health insurance system to reduce dependence on donors? If not, why not?

**Transformative Capacity of the Health System: Resource Supply (Workforce and Supply Systems)**

1. Has a continuous training system been designed to prepare health workers for crises and disasters? When? If not, why not?

2. Has mental health and psychosocial support been integrated for health workers after crises? When? If not, why not?

3. Have workforce distribution mechanisms been improved to include remote and hard-to-reach areas? When? If not, why not?

4. Has a national information system been developed to monitor the supply chain and prevent medicine stock-outs? When? If not, why not?

5. Is local manufacturing of essential medicines and supplies being supported? When? If not, why not?

6. Is technology being adopted to manage and distribute stocks based on actual needs and demand? When? If not, why not?

**4- Which public entity carries out the resilience assessment? Probe**

- Ministry of Health
- Ministry of Finance / Treasury
- National Institute of Health
- Payers (regional authorities, health insurance fund)
- Other non-ministerial public body
- Other (please specify)

**5. Based on your experience, what are the most significant gaps in the current resilience strategies? *Probe***

***Examples:***

1-Institutional accountability

2-Transparency of health system objectives and decision-making

3-Stakeholder participation in policy decisions

4-The capacity and organizational structures to address future shocks

5-Efficiency-enhancing measures implemented during "normal times" do not inadvertently create brittleness in the system

7-Other ________________________________________________

6. **What policy changes would you prioritize to strengthen health system resilience moving forward? *Probe***

**Examples:**

1. **Improving governance and empowering leadership**
2. **Strengthen surveillance system**
3. **Enhance human resources (frontline workers and include medicine students to bridge the gap of health providers shortage)**
4. **Community involvement**
5. **Innovative financing**
7. **How do you evaluate the health system's performance in real-time? *Probe***
8. **How do you evaluate the health system's readiness against any future shock? Did the health system in Yemen has a capacity to change its structure as a part of lesson learned? *Probe***
9. **What are your recommendations for enhancing health system resilience strategies in Yemen? *Probe***
